# Electric field distribution predicts efficacy of accelerated intermittent theta burst stimulation for late-life depression

**DOI:** 10.3389/fpsyt.2023.1215093

**Published:** 2023-08-01

**Authors:** Davin K. Quinn, Joel Upston, Thomas R. Jones, Benjamin C. Gibson, Tessa A. Olmstead, Justine Yang, Allison M. Price, Dorothy H. Bowers-Wu, Erick Durham, Shawn Hazlewood, Danielle C. Farrar, Jeremy Miller, Megan O. Lloyd, Crystal A. Garcia, Cesar J. Ojeda, Brant W. Hager, Andrei A. Vakhtin, Christopher C. Abbott

**Affiliations:** ^1^Department of Psychiatry and Behavioral Sciences, UNM, Albuquerque, NM, United States; ^2^Department of Psychology, University of New Mexico, Albuquerque, NM, United States; ^3^Department of Surgery, UNM, Albuquerque, NM, United States; ^4^Department of Psychiatry, Texas Tech University, El Paso, TX, United States; ^5^Mind Research Network, Albuquerque, NM, United States

**Keywords:** late-life depression, accelerated intermittent theta burst stimulation, neuronavigation, induced electric field, ventrolateral prefrontal cortex

## Abstract

**Introduction:**

Repetitive transcranial magnetic stimulation (rTMS) is a promising intervention for late-life depression (LLD) but may have lower rates of response and remission owing to age-related brain changes. In particular, rTMS induced electric field strength may be attenuated by cortical atrophy in the prefrontal cortex. To identify clinical characteristics and treatment parameters associated with response, we undertook a pilot study of accelerated fMRI-guided intermittent theta burst stimulation (iTBS) to the right dorsolateral prefrontal cortex in 25 adults aged 50 or greater diagnosed with LLD and qualifying to receive clinical rTMS.

**Methods:**

Participants underwent baseline behavioral assessment, cognitive testing, and structural and functional MRI to generate individualized targets and perform electric field modeling. Forty-five sessions of iTBS were delivered over 9 days (1800 pulses per session, 50-min inter-session interval). Assessments and testing were repeated after 15 sessions (Visit 2) and 45 sessions (Visit 3). Primary outcome measure was the change in depressive symptoms on the Inventory of Depressive Symptomatology-30-Clinician (IDS-C-30) from Visit 1 to Visit 3.

**Results:**

Overall there was a significant improvement in IDS score with the treatment (Visit 1: 38.6; Visit 2: 31.0; Visit 3: 21.3; mean improvement 45.5%) with 13/25 (52%) achieving response and 5/25 (20%) achieving remission (IDS-C-30 < 12). Electric field strength and antidepressant effect were positively correlated in a subregion of the ventrolateral prefrontal cortex (VLPFC) (Brodmann area 47) and negatively correlated in the posterior dorsolateral prefrontal cortex (DLPFC).

**Conclusion:**

Response and remission rates were lower than in recently published trials of accelerated fMRI-guided iTBS to the left DLPFC. These results suggest that sufficient electric field strength in VLPFC may be a contributor to effective rTMS, and that modeling to optimize electric field strength in this area may improve response and remission rates. Further studies are needed to clarify the relationship of induced electric field strength with antidepressant effects of rTMS for LLD.

## 1. Introduction

A significant percentage (10–15%) of the aging population experiences major depressive disorder, known as late-life depression (LLD), with negative impacts on functioning and quality of life (1). Mild cases can be addressed with education and counseling, while moderate to severe cases of LLD may require antidepressant medication or somatic therapies which can cause systemic and cognitive side effects (2). In particular, electroconvulsive therapy (ECT) is associated with risk of anterograde and retrograde memory loss (3), potentially compounding the cognitive deficits associated with neurodegenerative conditions, chronic medical conditions, and cerebrovascular disease. Efficacious treatments for LLD without risk of cognitive impairment are needed.

Repetitive transcranial magnetic stimulation (rTMS) is a FDA-approved therapeutic option for treatment-resistant depression that may be effective for LLD (4, 5). By generating electric currents in cerebral cortex through electromagnetic induction, rTMS is able to alter connectivity within and between large-scale brain networks involved in emotion regulation (6). When administered using various protocols such as 10 Hz, 1 Hz, or intermittent theta burst stimulation (iTBS), rTMS has demonstrated rates of up to 70% response and 40% remission in naturalistic studies (7, 8).

### 1.1. Atrophy in late-life depression may affect rTMS efficacy

Unfortunately, increased age has been associated with diminished rates of response and remission in multiple studies since the initial demonstrations of rTMS for depression (5). A 2022 systematic review of seven randomized trials and seven uncontrolled trials of rTMS for LLD [found significant variability in response rates (6.7–54.3%)] as well as parameters utilized (9). Suspected causes of reduced efficacy include vascular damage to structural white matter pathways along which rTMS effects propagate (10); the presence of common comorbidities in late-life depression, such as anxiety disorders (11), that are associated with lower remission rates with rTMS (12); and age-related cortical atrophy, which may require higher intensities of magnetic field strength to achieve adequate penetration. An early study of high frequency left dorsolateral prefrontal cortex (DLPFC) rTMS in LLD found the antidepressant response rate was greater in patients <65 years of age compared to those >65 (56% vs. 23%) (13), with the authors concluding that structural brain changes in persons with LLD contribute to reduced efficacy. Two more studies found no significant effect of rTMS treatment compared to placebo in persons with LLD (14, 15). Nahas et al. (16) showed that adjusting stimulation parameters for frontal atrophy resulted in an antidepressant effect in 27% of participants. Jorge et al. (17) performed a randomized sham-controlled trial of rTMS in persons with vascular depression and found that age and frontal gray matter atrophy were negatively correlated with response (16). Even in more contemporary studies using higher pulse counts, longer treatment durations and greater intensities, rTMS efficacy for LLD may be significantly diminished, such as in a recent small double-blinded rTMS trial that found 0% response in 10 patients receiving left unilateral excitatory stimulation alone (18). Heuristics to counteract effects of asymmetric atrophy such as adjusting motor threshold-based stimulation intensity according to scalp to cortex distance at the prefrontal target have been proposed and utilized (19, 20), but do not fully account for the effects of gyral thinning and sulcal widening on the induced electric field, and have generally not been used above the maximum stimulation intensity of 120% resting motor threshold.

### 1.2. Current targeting methods do not address electric field dose

Recent advances in accessibility of computational finite element modeling for use in noninvasive brain stimulation have enabled rapid calculation of the predicted induced electric field (*|E|*) of rTMS and correlation of its distribution and intensity with clinical and physiological outcomes. This capability provides a means for accurately and precisely assessing the effects of generalized and local atrophy on efficacy of rTMS in LLD. Electric field modeling has been used extensively in studies of the motor system, with strong correlations demonstrated between motor cortex *|E|*, coil-to-cortex distance, and motor threshold (21–23). There have been fewer clear results regarding *|E|* in the DLPFC for treatment of depression. A rTMS modeling study conducted in 121 patients from the Human Connectome Project database demonstrated high rates of inter-individual variability in *|E|* and its distribution, as well as in networks stimulated when rTMS is delivered to generic targets such as F3 (24). In a study of rTMS for smoking cessation by Caulfield et al. (25), *|E|* in the prefrontal cortex was shown to be significantly diminished compared to the motor cortex, with higher levels of stimulation needed in the prefrontal cortex (133% of motor threshold) to achieve the same *|E|* obtained in the motor cortex at 100% of threshold. A study by Deng et al. (26) of electric field strength in the middle, superior, and inferior frontal gyri of the DLPFC in 26 depressed patients receiving rTMS at F3 did not find a correlation with clinical outcomes. A recent study by Zhang et al. (27) of 12 patients receiving 3 weeks of left iTBS/right cTBS for depression found that the normal component of the electric field, not the tangential component or overall magnitude, was significantly correlated with antidepressant response. Finally, a comparison study was conducted by Deng et al. (28) between four targeting methods (5 cm rule, Beam F3, MRI-guided, and electric field-optimization) using pilot data from ten adolescents receiving 30 daily sessions of 10 Hz rTMS. Significant correlation was observed between *|E|* in the DLPFC and antidepressant response in patients receiving a full course of treatment. Of the above methods, the 5 cm rule method yielded the weakest field strength, and the Beam F3 method demonstrated significant variability.

To date, computational modeling has not been used to assess the relationship of *|E|* to clinical benefit with rTMS in an aged population. Therefore, we proposed and conducted a pilot study of accelerated fMRI-guided iTBS for patients with LLD and hypothesized that greater *|E|* measured at the personalized target would be associated with greater antidepressant response.

## 2. Methods

This was an unblinded, single-arm, prospective cohort study of accelerated fMRI-targeted iTBS conducted in 25 patients aged 50 and older with a diagnosis of major depressive disorder. This protocol was reviewed and approved by the UNM Health Sciences Center Human Research Review Committee (HRRC #19–531).

### 2.1. Recruitment

Recruitment took place through the UNM Treatment Resistant Depression Clinic, Geriatric Psychiatry Clinic, TMS Service, and ECT Service. All participants from the various clinics were referred for consideration of rTMS treatment for major depression, having failed various therapeutics such as oral antidepressants, esketamine, ECT, or traditional rTMS. Participants were screened *via* phone for inclusion and exclusion criteria.

### 2.2. Inclusion/exclusion criteria

To be enrolled in the study, participants met the following inclusion criteria: 1) ages 50–79, 2) diagnosis of major depressive disorder of at least 6 months’ duration preceding study entry, confirmed by two independent board-certified psychiatrists according to DSM-5 criteria, 3) four or more adequate trials of antidepressants in the current episode, and 4) score of 10 or higher on the Quick Inventory of Depressive Symptomatology (16 item) (Self-Report) (QIDS-SR-16) at time of study entry. Exclusion criteria included: 1) history of seizure, 2) history of a major neurocognitive disorder or central nervous system disorder diagnosis, 3) implanted ferromagnetic material or contraindication to obtaining MRI, 4) pregnancy, 5) current incarceration, 6) inability to complete the protocol, 7) medical instability resulting in hospitalization or emergency department visit within the past month, and 8) psychotropic medication change or treatment with electroconvulsive therapy within the month preceding study entry.

### 2.3. Visit 1 assessment

After screening and consent, participants underwent demographic survey (age, sex, socioeconomic status, educational attainment, ethnicity, race, and handedness); assessment of depression history and treatment; mood and anxiety symptom assessment with the Inventory of Depressive Symptomatology for Clinicians (IDS-C-30, primary outcome measure); Generalized Anxiety Disorder-7 (GAD-7); Snaith-Hamilton Assessment of Pleasure Seeking for Clinician Administration (SHAPS-C); Temporal Experience of Pleasure Scale (TEPS); and the Behavioral Inhibition System/Behavioral Approach System Scale (BIS/BAS). Select domains of cognition were assessed with the Delis-Kaplan Executive Function Scale (DKEFS), Wechsler Adult Intelligence Scale (WAIS), and Hopkins Verbal Learning Test-Revised (HVLT). These instruments were chosen in line with prior study protocols combining imaging and neuromodulation (29, 30).

### 2.4. MRI

At the baseline visit, participants underwent structural and resting-state functional magnetic resonance imaging (MRI) on a 3 T Siemens Prisma scanner. High-resolution T_1_- and T_2_-weighted structural images and two 6-min runs of resting-state functional MRI (rsfMRI) were obtained. For structural scans: repetition time (TR) = 2,530 milliseconds (ms), echo time (TE) = 1.64, 3.5, 5.36, 7.22, 9.08 ms, Inversion time (TI) = 1,200 ms, flip angle = 7.0°, slices = 192, field of view = 256, matrix 256 × 256, voxel size = 1.0 × 1.0 × 1.0 millimeter (mm). For resting-state scans: TR = 480 ms (multiband acceleration factor of 8), TE = 29 ms, flip angle (FA) = 75°, slices = 192, voxel size = 2.0 × 2.0 × 2.0 mm. The T_1_ was preprocessed by parcellating with Freesurfer 6.0.0 and then aligned to rsfMRI data (31). The rsfMRI was preprocessed using AFNI’s recommended pipeline (example 11) afni_proc.py with AFNI 20.2.18 (32). The first four volumes of each run were dropped and each run was aligned and despiked, slice time corrected, distortion corrected, warped to Montreal Neurological Institute (MNI) space, blurred with a 4 mm full width at half maximum (FWHM) Gaussian kernel, and scaled to a mean of 100. Nuisance signals were regressed and outlying volumes censored, and the runs were concatenated.

### 2.5. Targeting

Resting-state fMRI analysis and determination of neuronavigation targets were based on the published method of Ning et al. (33). The use of resting-state fMRI to identify targets within the DLPFC is built on a growing body of lesion and imaging work demonstrating the SgCC as a critical region mediating depressive symptomatology (34, 35). fMRI studies particularly by Fox et al. (36, 37) have shown that the degree of intrinsic anticorrelated activity between the DLPFC and SgCC at the target is a predictor of response to rTMS. More recent studies have demonstrated that distance of the stimulated target from the maximum anticorrelated target correlates with response to treatment (36, 38, 39). Our seed region was defined using the Brainnetome atlas region corresponding to the SgCC (187, 188), and the bounding search region within the DLPFC in each hemisphere was created from Brainnetome regions (15, 16, 19, 20, 21, 22) making up Brodmann areas 9 and 46 (40). Functional connectivity was measured by correlating time-series data from the pre-processed resting-state fMRI data for the seed region with each voxel in the search regions. A mask was created with the maximum anticorrelated voxel in the search region. Structural T_1_ images and the functional mask were then exported to the Localite neuronavigation system for registration during stimulation sessions.

### 2.6. Stimulation

The 25 participants each received a total of 45 sessions (five sessions/day, nine weekdays) of iTBS to the cortical target with a MagPro X100 equipped with a Cool-B70 coil (Magventure Inc., Alpharetta, GA). The right DLPFC was chosen as the initial target region given its potential efficacy for depressive and anxious symptoms (41, 42), and based on earlier work showing that iTBS to this area can improve cases of depression that do not respond to iTBS to the left DLPFC (43). Co-registration of the MRI data in the Localite neuronavigation system was performed with head landmarks at the nasion and bilateral tragus. The mask with the functional target was overlaid on the structural images and projected orthogonally to the nearest scalp surface for coil positioning. Coil rotation at the scalp projection was specified as 45° from midline, with the coil handle pointing posteriorly. Coil tilt was maintained tangential to the plumb line from scalp projection to brain target. Deviation from target during iTBS was monitored and the coil repositioned for any displacements greater than three millimeters. In each session, 1800 pulses were delivered in 60 trains of 10 triplet bursts (pulse frequency 50 Hz, burst frequency 5 Hz), 2 s train duration, and 8 s intertrain interval in accordance with recently published accelerated iTBS protocols by Cole et al. (44). Pulses were delivered at 120% of resting motor threshold (RMT), defined as the minimum amount of energy to obtain five out of 10 motor evoked potentials with peak to peak amplitude of at least 50 uV in the abductor pollicis brevis muscle on electromyography, in accordance with parameters from the iTBS noninferiority study by Blumberger et al. (45). If patients could not tolerate 120% of RMT due to scalp discomfort, the highest tolerable stimulation intensity up to 120% RMT was delivered. Each iTBS session was separated by 50 min, based on prior work demonstrating this time frame as the optimal recovery time between sessions for accelerated protocols (46).

### 2.7. Visit 2 and 3 assessments

After 15 sessions participants repeated all behavioral assessments as this corresponds to the timeframe for mid-course evaluation in a typical clinical rTMS course. They then received 30 more sessions. If there was minimal improvement (<10%) noted in IDS-C-30 score at Visit 2 or development of intolerable side effects, the participant was switched to stimulation of the left hemisphere for the remainder of treatment, in line with clinical practice. The day following completion of the 45th session, participants repeated behavioral assessments and cognitive testing (Visit 3). At 1 month and 2 months following the protocol, the subjects were contacted by phone and assessed with the IDS-C-30.

### 2.8. Statistical analysis

Means and standard deviations for demographics and baseline behavioral and cognitive measures were calculated in SPSS Statistics 26 (IBM; Armonk, NY). Repeated-measures analyzes of variance and effect sizes expressed as partial eta squared (*η*_p_^2^) were calculated for Visit 1, 2, and 3 behavioral and cognitive outcomes using R v. 4.1.3 (R Foundation; Vienna, Austria).

### 2.9. Electric field modeling

Using the T1 and T2 weighted images within Simulation of Non-Invasive Brain Stimulation (SimNIBS) (47) a segmented 10-tissue head model was created and a simulated coil placed at the personalized target of each participant. The modeled coil orientation was defined as tangential to the scalp and rotated 45° from midline with the coil handle pointing posteriorly. A model based on a quasi-static approximation of Maxwell’s equations was then solved for the vectorwise induced electric field (E), which is then scaled by the actual intensity of the individual stimulation delivered (% of maximum device output). To simplify calculations and the emphasis of directionality of the electric field, the magnitude of the induced electric field was calculated(|E|). This induced electric field measure was averaged in each parcellated region across the whole brain using the Human Connectome Project multimodal atlas parcellation (48) (HCP-MMP) for each subject to create regional induced electric field measures. Electric field analysis was restricted to areas that received significant field magnitude [defined as any area above half of the maximum brain average field (|E_max_|/2) or above half of the maximum induced standard deviation (|E_max,sd_|/2)], based on the entire cohort’s |E|. This restricted the analysis to 11 regions in the HCP-MMP atlas. Due to segmentation issues only 23 of 25 participants’ electric fields were included in the analysis.

## 3. Results

### 3.1. Baseline characteristics

Baseline characteristics of the study population are displayed in Table 1. The participants were predominantly female (22 of 25, 88%) and Caucasian (23 of 25, 92%), consistent with composition of the referring clinics. Comorbid psychiatric diagnoses (e.g., generalized anxiety disorder, GAD; posttraumatic stress disorder, PTSD) were present in 44% of participants. Treatment resistance was high, with participants on average having trialed nine medications prior to study entry, and 32% having previously trialed ECT.

**Table 1:** Demographic and clinical characteristics of the study population (*N* = 25).

Variable	Value
Age, years	65 ± 7
Sex	
Male	3 (12)
Female	22 (88)
Education (years)	6.5 ± 1.4
BMI	29.6 ± 7.3
Ethnicity	
Non-Hispanic	23 (92)
Hispanic	2 (8)
Race	
Caucasian	23 (92)
Other	2 (8)
Comorbid psychiatric diagnoses	
None	14 (56)
GAD	9 (36)
PTSD	6 (24)
Other	2 (8)
Episode duration (months)	202.0 ± 196.5
Lifetime duration (years)	42.7 ± 15.7
History of ECT	
Yes	8 (32)
No	17 (68)
Test of premorbid function scaled score	108.5 ± 21.7
Family history	
Yes	21(84)
No	4(16)
Number of failed antidepressant trials	9.3 ± 6.0

Values are number (%) or mean ± standard deviation.

### 3.2. Side effects

The most common reported side effects were scalp discomfort (68%), headache (48%), fatigue (40%), and sleep disruption (36%). All 25 participants completed all assessments for Visits 1, 2, and 3, and no participants discontinued involvement in the study. The average intensity of stimulation tolerated was 114.9% of RMT, with five subjects not able to tolerate the full dose of 120% of RMT.

### 3.3. Depression

**Figure 1:**
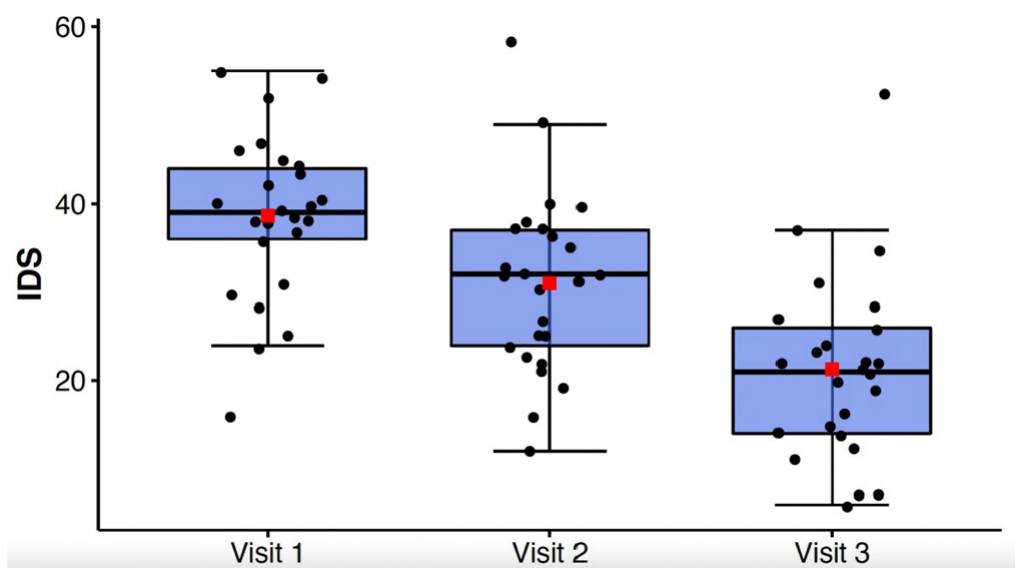
Box-whisker plot of IDS-C-30 scores at study Visits 1, 2, and 3. Center box lines indicate medians, red squares indicate means. Shaded box areas indicate 25th–75th percentile values. Bars indicate 1.5x interquartile range.

Table 2 contains means and standard deviations for each behavioral and cognitive measure as well as *p* values and effect sizes. Assumptions of normality and sphericity were met for the primary outcome measure of depressive symptoms, the IDS-C-30, and most behavioral and cognitive secondary outcome measures. For certain secondary outcome measures such as the GAD-7, Letter Fluency, and Color Word Score where assumptions of sphericity were not met, Greenhouse–Geisser correction was applied to the repeated measures ANOVA results. Mean depression scores for the entire cohort as measured by the IDS-C-30 improved significantly from Visit 1 to Visit 3 (Visit 1: 38.6 +/− 9.31; Visit 2: 31.0 +/− 10.2; Visit 3: 21.3 +/− 10.4; *F*(2,48) = 62.88, *p* = < 0.0001, *η*_p_^2^ = 0.72) (Figure 1). Clinical response, defined as > = 50% improvement in depression score, was achieved in 13 out of 25 subjects (52%) by Visit 3, and remission, defined as IDS-C-30 = < 12, was achieved in 5 out of 25 subjects (20%). Post-hoc t-tests with Bonferroni correction confirmed significant decreases in depression scores between Visit 1 and 2 (*t*(24) = 5.90, *p* < 0.0001), Visit 1 and 3 (*t*(24) = 9.64, *p* < 0.00001), and Visit 2 and 3 (*t*(24) = 6.41, *p* < 0.00001). An exploratory analysis of long-term effects was undertaken with follow-up IDS-C-30 assessment *via* phone call to all participants at 1 month and 2 months after treatment. Two participants were not able to be reached for 1 month assessment; at three-month follow-up, five participants were not able to be reached; these were the only missing data points in the cohort. Mean IDS-C-30 scores and standard deviations at one-month follow-up were 22.4 +/− 15.0. At three-month follow-up, mean IDS-C-30 score and standard deviation were 25.5 +/− 12.3.

**Table 2:** Means and standard deviations (in parentheses) for primary and secondary behavioral outcome variables and cognitive assessments at Visits 1, 2, and 3.

	Visit 1	Visit 2	Visit 3	*F*	df	*p*	*η*_p_^2^
IDS-C-30^a^	38.64 (9.31)	30.96 (10.2)	21.28 (10.41)	62.88	2,48	**<0.0001**	0.72
GAD-7	9.72 (5.46)	9.00 (4.81)	5.6 (4.81)	12.74	1.57,37.6	**<0.001**	0.35
TEPS	60.44 (11.23)	65.52 (11.37)	68.16 (11.10)	7.85	2,48	**0.001**	0.25
SHAPS-C	38.8 (9.05)	34.80 (8.31)	32.32 (8.91)	10.47	2,48	**<0.001**	0.3
BAS drive	8.80 (2.81)	10.20 (2.47)	10.00 (2.24)	4.51	2,48	**0.016**	0.16
BAS fun seeking	8.80 (2.55)	9.48 (2.31)	9.24 (2.42)	1.95	2,46	0.15	0.07
BAS reward resp	14.80 (2.12)	15.44 (1.64)	16.32 (1.93)	7.21	2,48	**0.002**	0.23
BIS	24.20 (3.15)	23.88 (3.87)	22.52 (3.50)	11.9	2,46	**<0.0001**	0.33
DKEFS							
Letter fluency total correct scaled score	12.17 (3.57)	12.6 (3.27)	12.52 (3.45)	2.19	1.6,36.7	0.14	0.087
Category fluency total correct scaled score	11.35 (3.34)	11.36 (3.11)	11.24 (4.14)	0.07	2,44	0.935	0.003
Category switching total correct scaled score	11.39 (3.64)	12.00 (3.42)	12.46 (3.28)	1.49	2,42	0.24	0.066
Category switching accuracy scaled score	11.04 (3.70)	11.80 (3.16)	12.04 (3.16)	1.26	2,42	0.29	0.057
Color-word condition 1 color scaled score	8.96 (3.83)	9.12 (2.99)	9.60 (3.20)	2.23	2,44	0.12	0.092
Color-word condition 2 word scaled score	9.48 (3.19)	9.12 (2.44)	9.04 (2.75)	0.22	2,44	0.81	0.01
Color-word condition 3 inhibition scaled score	9.70 (3.90)	10.12 (2.98)	10.20 (3.48)	0.84	1.44,31.76	0.41	0.037
Color-word condition 4 inhibition switch scaled score	10.18 (3.26)	10.28 (2.94)	10.60 (3.11)	0.54	1.45,30.45	0.53	0.025
Color-word composite scaled score	9.48 (3.38)	9.28 (2.51)	9.64 (2.72)	1.04	1.55,34.11	0.35	0.045
HVLT							
Total recall correct T score	46.63 (13.37)	49.40 (8.35)	47.84 (11.34)	0.90	2,46	0.41	0.038
Delayed recall correct T score	48.17 (11.50)	49.60 (9.17)	49.00 (10.57)	0.21	2,46	0.81	0.009
Retention T score	52.71 (13.22)	50.04 (9.97)	51.00 (10.68)	0.40	2,46	0.67	0.017
Recognition discrimination index T score	47.63 (7.92)	46.44 (9.95)	46.60 (10.79)	0.35	2,46	0.71	0.015
WAIS							
Digit span scaled score	11.76 (2.61)	11.33 (2.35)	12.18 (2.35)	0.96	2,18	0.40	0.097

a Indicates primary outcome measure.

IDS-C-30, Inventory of Depressive Symptoms-30-Clinician; GAD-7, Generalized Anxiety Disorder-7; TEPS, Temporal Experience of Pleasure Scale; SHAPS-C, Snaith-Hamilton Pleasure Scale-Clinician; BAS, Behavioral Approach System; BIS, Behavioral Inhibition System; DKEFS, Delis-Kaplan Executive Function System; HVLT, Hopkins Verbal Learning Test; WAIS, Wechsler Adult Intelligence Scale.

### 3.4. Switching

A total of six patients switched to left hemisphere treatment during the protocol due to lack of at least 10% improvement in the IDS-C-30 at Visit 2. Of the subjects that switched, by Visit 3 none met criteria for remission, two met criteria for response, two met criteria for partial response (25–50% improvement), and two patients did not respond (Visit 1: 38.8 +/− 9.24; Visit 2: 38.7 +/− 9.69; Visit 3: 26.0 +/− 9.72).

### 3.5. Anxiety

**Figure 2:**
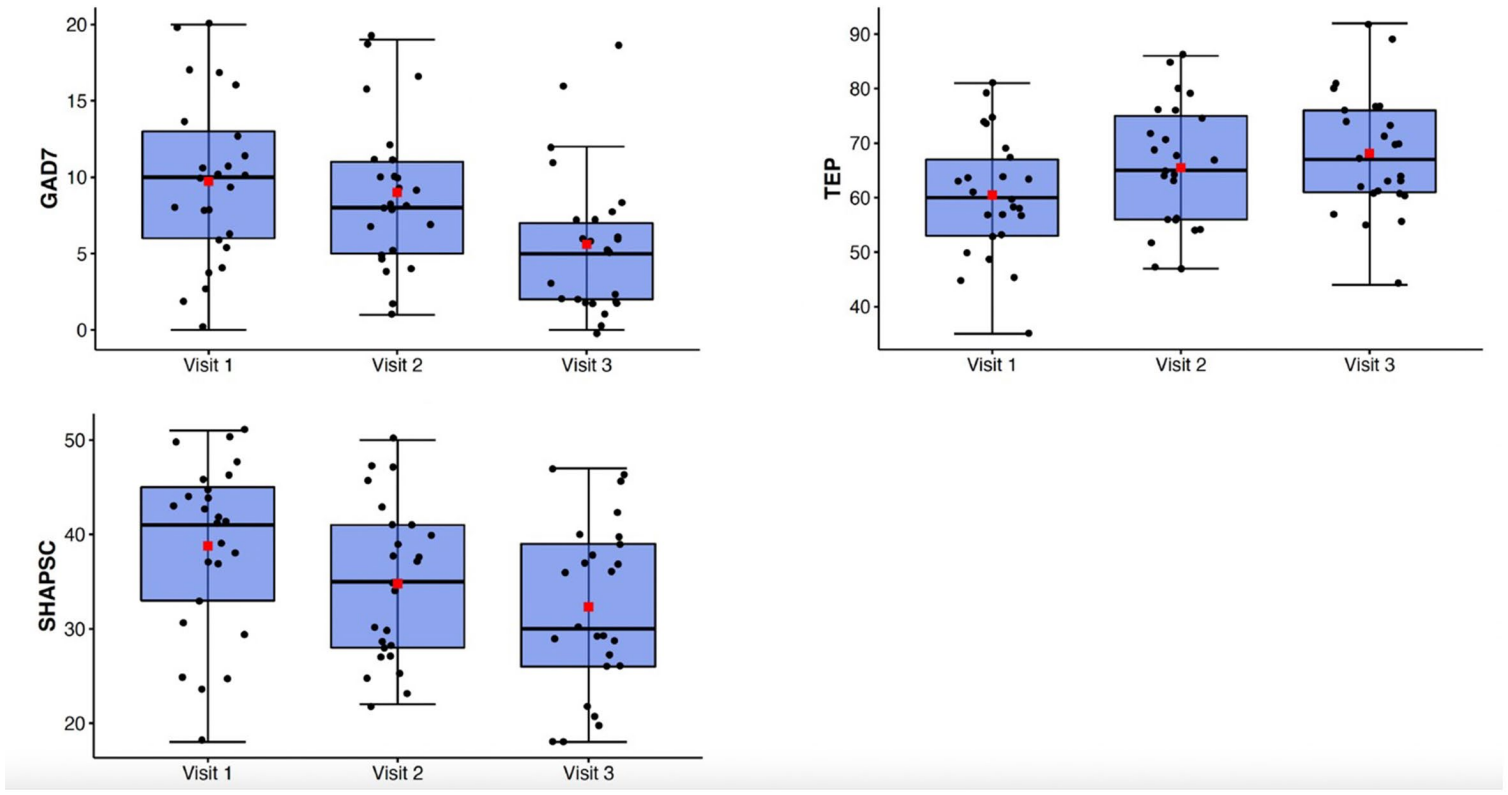
Top: box-whisker plots of GAD-7, TEPS, SHAPS-C at study Visits 1, 2, and 3. Center box lines indicate medians, red squares indicate means. Shaded box areas indicate 25th–75th percentile values. Bars indicate 1.5x interquartile range.

**Figure 3:**
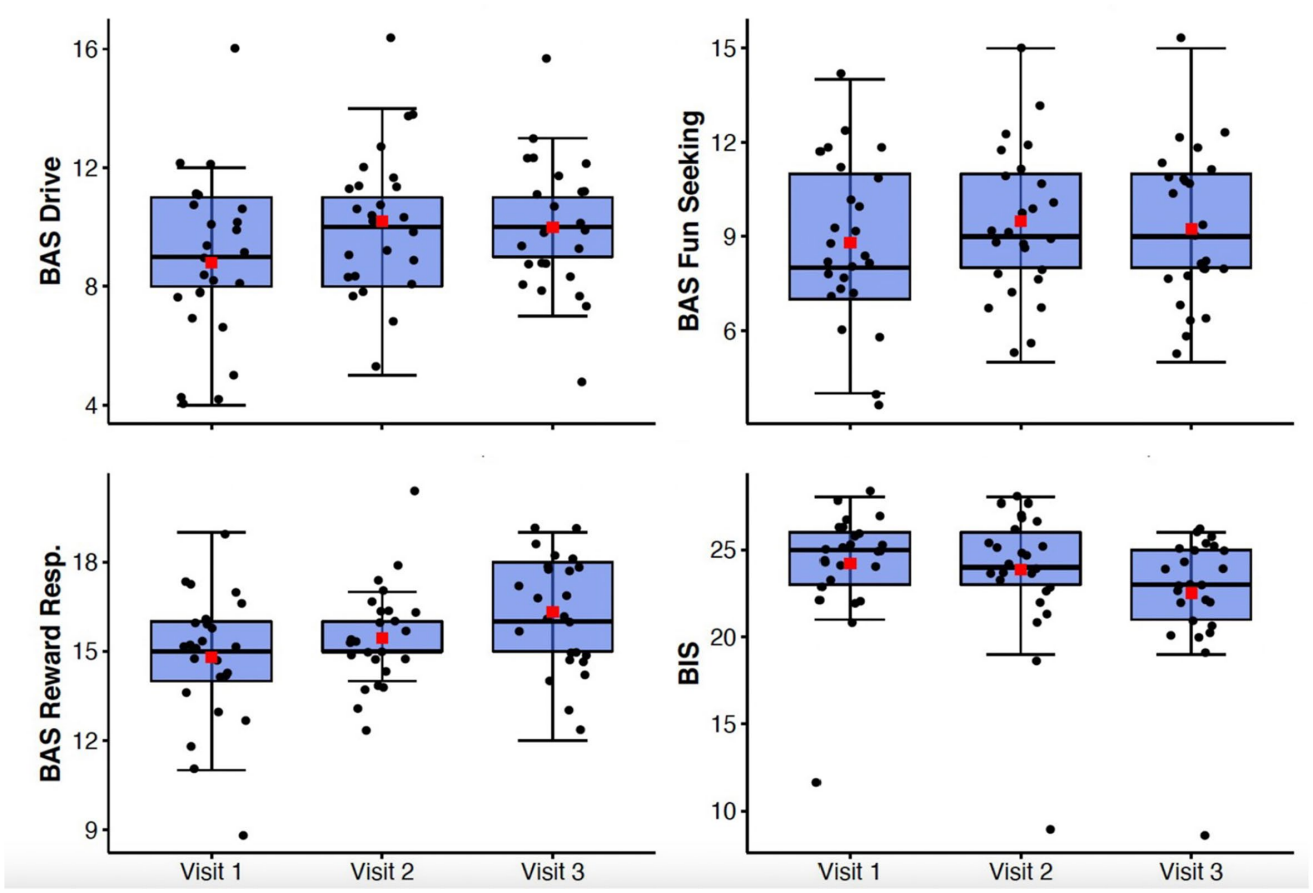
Box-whisker plots of BIS/BAS subscale scores at study Visits 1, 2, and 3. Center box lines indicate medians, red squares indicate means. Shaded box areas indicate 25th–75th percentile values. Bars indicate 1.5x interquartile range.

Generalized anxiety symptoms as measured by the GAD-7 declined significantly from Visit 1 to Visit 3 (*F*(1.57, 37.6) = 12.74, *p* < 0.001, *η*_p_^2^ = 0.35) (Figure 2). Behavioral inhibition as measured by the BIS/BAS demonstrated improvement with treatment (*F*(2,48) = 11.9; *p* < 0.0001; *η*_p_^2^ = 0.33) (Figure 3).

### 3.6. Anhedonia

There were significant improvements observed in anhedonia symptoms from Visit 1 to Visit 3 as measured by the TEPS (*F*(2,48) = 7.85, *p* = 0.001, η_p_^2^ = 0.25) and the SHAPS-C (*F*(2,48) = 10.47, *p* < 0.001, *η*_p_^2^ = 0.3) (Figure 2). Behavioral approach as measured by the BIS/BAS also demonstrated significant changes, with increases in Reward Responsivity (*F*(2,48) = 7.21; *p* = 0.002; η_p_^2^ = 0.23) and Drive (*F*(2,48) = 4.51; *p* = 0.016; *η*_p_^2^ = 0.16) (Figure 3). After Bonferroni correction for multiple comparisons, the findings for the Drive subscale were no longer significant.

### 3.7. Cognition

There were no significant changes in any of the cognitive domains tested, including short term memory (HVLT-R), attention (WAIS), and executive function (DKEFS) from Visit 1 to Visit 3 (see Table 2).

### 3.8. Target distribution

**Figure 4:**
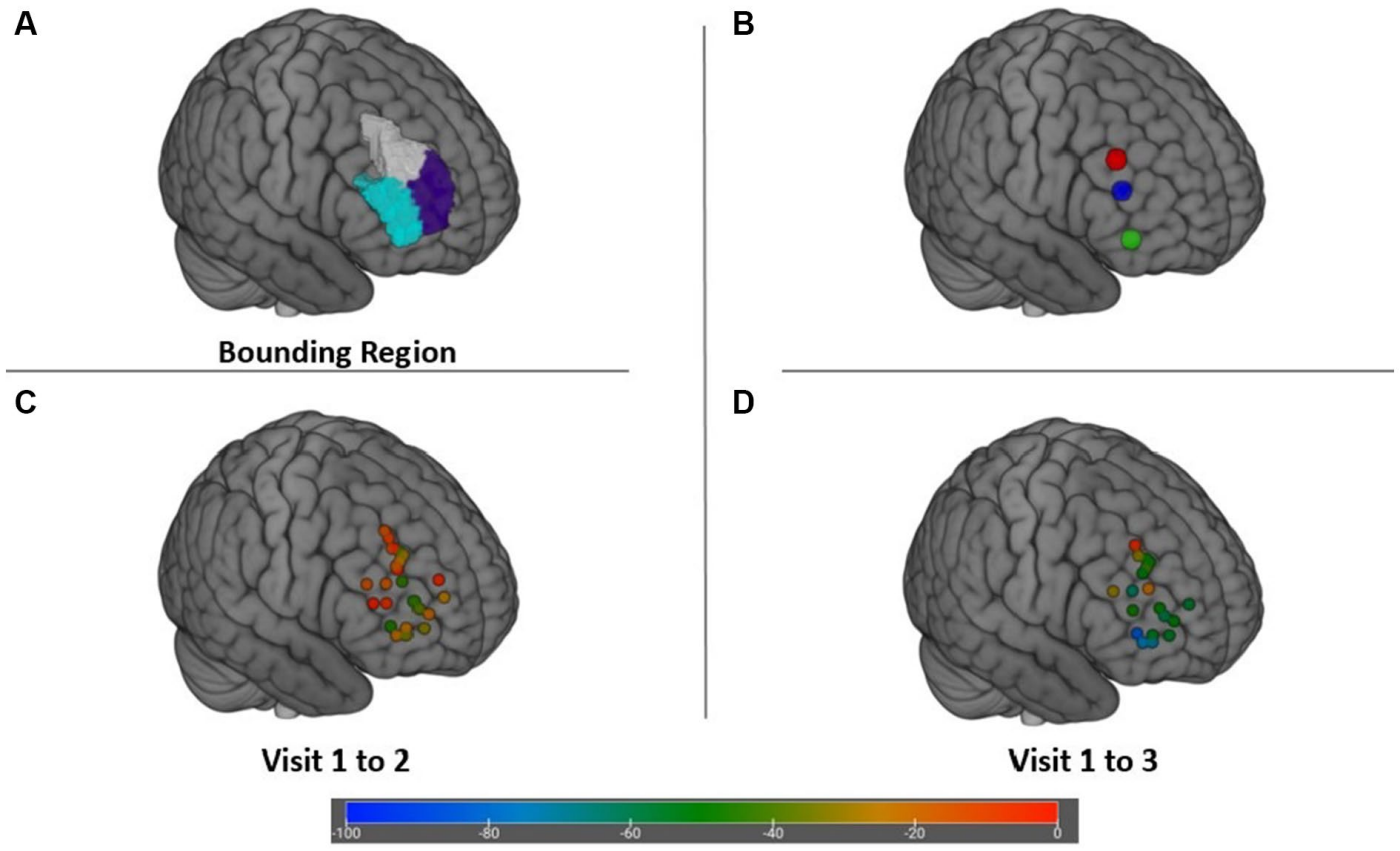
**(A)** Bounding search region within the DLPFC for targets maximally anticorrelated with the SgCC. **(B)** Cortical locations of scalp target F4 (X + 47 Y + 34 Z + 38) (red), depression network connectivity target from Siddiqi et al. (35) (X + 48 Y + 38 Z + 23) (blue), and posterior Brodmann area 47 (X + 46 Y + 43 Z-3) (green). **(C)** Change in IDS-C-30 from Visit 1 to Visit 2 achieved at each target. **(D)** Change in IDS-C-30 from Visit 1 to Visit 3 for participants receiving all 45 sessions to the right DLFPC.

The DLPFC target search region in Figure 4A and spatial distribution of the right DLPFC targets as well as their associated efficacy at Visit 2 and Visit 3 are displayed in Figures 4C,D. Also portrayed in Figure 4B are the cortical position of scalp location F4, as well as the right anterolateral anticorrelated network connectivity target identified by Siddiqi et al. *via* aggregative analysis of multiple imaging and brain stimulation data sets (35, 49). Degree of anticorrelation of the DLPFC targets with the SgCC was not significantly associated with change on the IDS-C-30 (*r* = −0.002; *p* = 0.28); however, anticorrelation between the DLPFC targets and SgCC showed a moderate positive correlation with increasing age (*r* = 0.39, *p* = 0.05), i.e., anticorrelation magnitude decreased as age increased.

### 3.9. Electric field distribution

**Figure 5:**
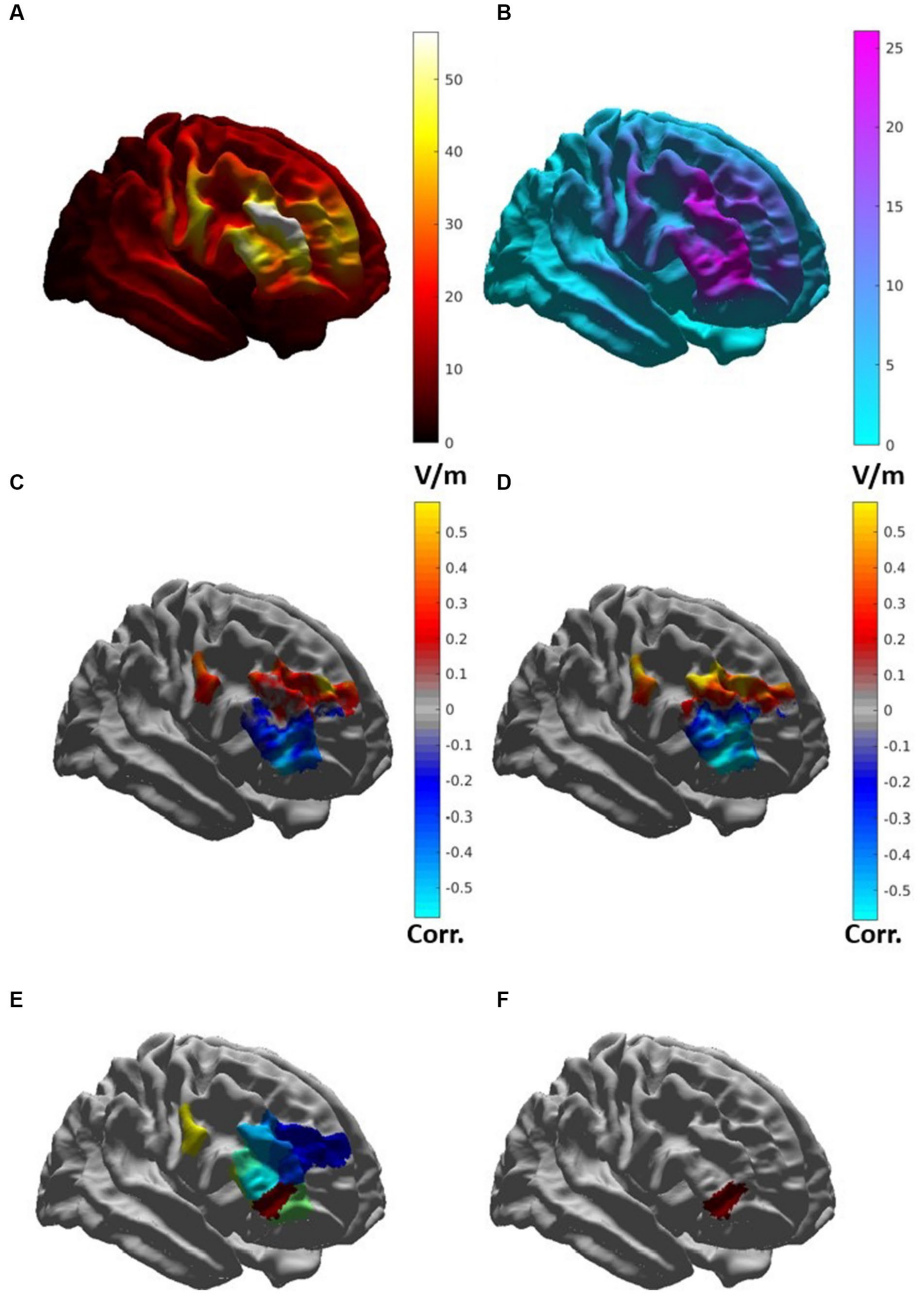
**(A)** Map of average induced electric field magnitude |*E*| (in V/m) for 23 patients. **(B)** Map of standard deviation of |*E*| (in V/m), indicating areas with high degree of variability. **(C)** Map of correlation between |*E*| and change in IDS-C-30 from Visit 1 to Visit 2 in brain regions included for analysis. Cool colors indicate areas of negative correlation, warm areas indicate areas of positive correlation. **(D)** Map of correlation between |*E*| and change in IDS-C-30 from Visit 1 to Visit 3 in participants receiving all 45 sessions to the right DLFPC. **(E)** HCP-MMP regions included for electric field analysis. **(F)** Posterior rostral Brodmann area 47 (p47r).

**Figure 6:**
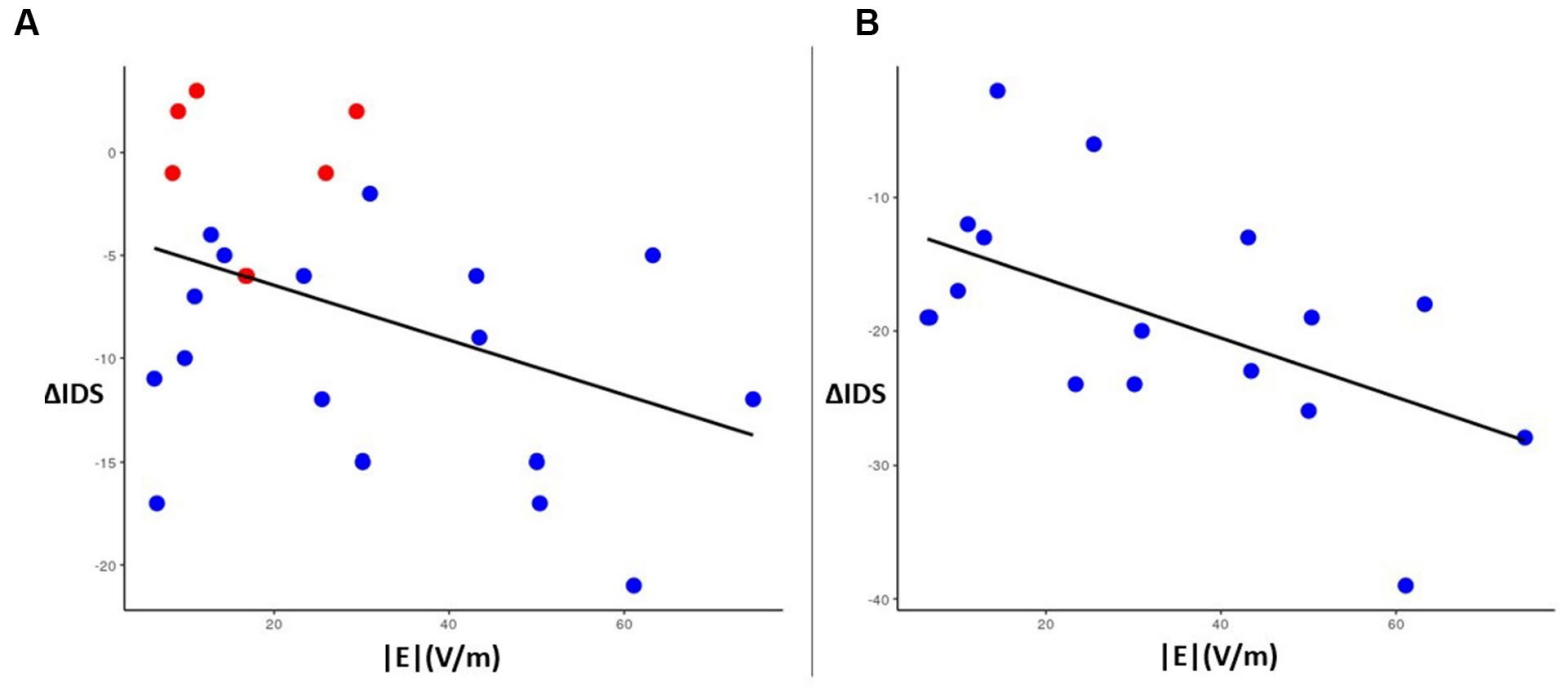
Left: correlation plot of magnitude |*E*| (x-axis) in region p47r with change in IDS-C-30 score (y-axis) between Visit 1 and Visit 2 for all participants (*N* = 23) (*r* = −0.41, *p* = 0.05). Red dots indicate the six participants who switched from right side to left side stimulation for Visit 2 to Visit 3. Right: correlation plot of |*E*| (x-axis) in region p47r with change in IDS-C-30 score between Visit 1 and Visit 3 for participants receiving all 45 sessions to the right DLFPC (*N* = 17) (*r* = −0.56, *p* = 0.02).

The average induced electric field for all participants was distributed broadly across the right frontal lobe, with regions of greatest *|E|* found in the middle frontal gyrus and inferior frontal gyrus (Figure 5A). *|E|* at the target (|*E_target_|*) for each patient was not significantly associated (*p* > 0.1) with change in IDS score, nor was |*E_target_|* correlated with the simulated electric field magnitude at the motor cortex (scalp location C3). In whole-brain analysis, negative correlations were observed between *|E|* and change in IDS-C-30 between Visit 1 and 2 and Visit 1 and 3 (i.e., higher field magnitude associated with greater reduction in IDS score and antidepressant benefit) in anterior and lateral regions, i.e., Brodmann areas 10, 47, and 45 (Figures 5B,C). Positive correlation between *|E|* and change in IDS-C-30 (i.e., higher field magnitude associated with less reduction or even increase in IDS score) was observed in posterior dorsolateral, dorsomedial, and motor regions. Of all areas meeting criteria for inclusion in electric field analysis, only posterior rostral Brodmann area 47 (p47r; Figure 5D) was significantly associated with change in IDS-C-30 score. The degree of correlation was moderate between *|E|* in p47r and change in IDS-C-30 from Visit 1 to 2 (*r* = −0.41, *p* = 0.05). In participants who received all 45 stimulation sessions to the right hemisphere, the degree of correlation was strong between *|E|* in p47r and change in IDS-C-30 from Visit 1 to 3 (*r* = −0.56, *p* = 0.02) (Figure 6). After controlling for false discovery rate, *p_fdr_* = 0.12.

## 4. Discussion

In this open-label, single-arm pilot study, accelerated fMRI-guided iTBS significantly improved depressive and anxious symptoms in 25 patients with LLD. The protocol itself was well-tolerated, with no participants discontinuing treatments early. The most common side effects were scalp discomfort, mild headache, sleep disruption, and fatigue. There were no serious adverse events and no significant changes on any of the cognitive measures obtained, indicating that accelerated iTBS at clinical stimulation intensities (110–120%) is a safe form of neuromodulation even in a population at increased risk of cognitive side effects. A very large effect was observed in the primary outcome measure, the IDS-C-30, with a mean 17-point reduction in depressive symptoms observed by the end of 45 iTBS sessions over 9 days, equating to a 52% response rate and 20% remission rate. Large effects on generalized anxiety levels and anhedonia symptoms were also demonstrated in the cohort. Our results highlight the rapidity of clinical benefit seen with accelerated protocols (44), in which response and remission can be achieved in 10 days or less compared to six to eight weeks with traditional once-daily clinical rTMS.

### 4.1. Utility of right iTBS for depression

This is the first rTMS study for LLD to target the right DLPFC with iTBS, an excitatory paradigm, and the first to use individualized fMRI guidance to the right DLPFC. The response rate of 52% is comparable to those described in uncontrolled non-accelerated studies of rTMS to the left DLPFC in general adult populations (7, 8), adding to the growing evidence base supporting targeting the right DLPFC with iTBS as an effective alternative strategy for treating depression (43, 50). These positive results run counter to a long-standing theory of hemispheric asymmetry of emotion regulation in the rTMS literature which posits that neuromodulation of the right DLPFC should be inhibitory in nature (i.e., 1 Hz) to be effective for depression (51). Recent work synthesizing results from multiple imaging and neuromodulation cohorts supports a conceptualization of the hemispheres as having relatively symmetric anticorrelated nodes in the DLPFC with similar relationships to depressive symptoms and treatment response to rTMS (35). Based on our clinical experience, up to 50% of patients will fail to respond to left DLPFC stimulation alone, highlighting the need for an accelerated iTBS protocol in the right hemisphere with an acceptable rate of benefit.

### 4.2. Clinical and stimulation factors influencing efficacy

Our remission rate of 20% was lower than described by Cole et al. (44) in the SAINT protocol study delivering accelerated fMRI-guided iTBS to the left DLPFC. They achieved 84% remission in an uncontrolled single-arm design and 79% remission in the active group in a subsequent double-blind trial (52). We believe our lower remission rate reflects several potential factors that may have relevance for wider use of accelerated protocols. First, the SAINT protocol delivered 50 sessions of iTBS in 5 days at 90% of motor threshold, whereas our protocol was 45 sessions over 9 days at 120% of motor threshold; the number, pace of acceleration, and dose of treatments may have had effects on the overall rate of clinical response, and not simply on the rapidity of improvement. Second, the SAINT protocol enrolled a younger population of adults (average age 45 vs. 65 in our study), with lower average number of medication trials (5 vs. 9) and fewer ECT-experienced patients (0% vs. 32%). There was a high degree of comorbidity in our cohort, with 44% of participants with a secondary psychiatric condition, especially anxiety disorders. Each of these factors has been independently associated with lower rates of response and remission (7, 16, 17). Third, our protocol targeted the right DLPFC with iTBS, a less studied paradigm for depression than left iTBS, 10 Hz, or right 1 Hz inhibitory approaches. While right DLPFC iTBS has been shown to have benefit for patients who fail to respond to left side stimulation (43, 50), its approximate effect size for depression is not yet established, thus it is a possibility that iTBS to the right DLPFC is overall less efficacious for depression compared to the left DLPFC.

A fourth factor that may have contributed to lower response and remission rates is the accuracy of the functional targeting method used in our study. The maximum anticorrelated target in the DLPFC is theorized to be the subregion through which rTMS may most robustly modulate SgCC activity, which has been extensively linked to depressive symptomatology (34, 36). Several algorithms for targeting have been published, with varying degrees of incorporation of normative data sets and varying findings with regard to stability of targets (33, 44, 53). The voxel-based method utilized in this study has been critiqued for the level of variability in its generated targets, with clustering and network connectivity analyzes currently being more favored (33, 53). It is possible that using one of these alternative methods may have obtained better outcomes. However, there has not been a head-to-head trial of one fMRI-based targeting method versus another, nor has there been a definitive controlled trial of fMRI-based targeting versus scalp-based targeting. In addition, a recently published network connectivity target for depression in the right hemisphere obtained from multiple imaging and neuromodulation datasets (35, 49) (Figure 4B) fell within our target bounding region (Figure 4A) and near the center of the average induced electric field (Figure 5A), suggesting that while our targets may have been distributed diffusely through the bounding region, they were likely not inaccurate in general. The fMRI targeting pipeline used in our pilot study was selected based on its feasibility of implementation using published information; its use of freely available software; its basis on each patient’s scan results and not group averages to compute the maximum anticorrelated node within the specified bounding region; and its ability to generate targets within 24 h of image acquisition. That there are multiple available targeting methods for fMRI-guided rTMS with different strengths and weaknesses highlights the need for studies comparing clinical efficacy of these methods.

### 4.3. Antidepressant effects in VLPFC

Of importance to the discussion of efficacy is our demonstration that *|E|* magnitude associates with clinical outcome for rTMS, the first such study in the right hemisphere. Greater *|E|* magnitude in anterior dorsolateral and ventrolateral regions was associated with greater antidepressant effect and was mirrored by the finding that greater *|E|* in posterior DLPFC was associated with less benefit. This anterior–posterior gradient agrees with theoretical work (35, 38) that posits an anterolateral anticorrelated node adjacent to correlated regions, with clinical benefit increasing the closer the target is to the anticorrelated node. We believe our findings provide evidence indicating that delivery of sufficient *|E|* to the functionally anticorrelated target is necessary for clinical response.

In addition, we note that the most impactful electric field effects on antidepressant outcome were not found in the targeted DLPFC, but in Brodmann area 47 (BA 47), a region categorized as ventrolateral prefrontal cortex (VLPFC), inferior frontal gyrus (IFG), and pars orbitalis. BA 47 has been implicated in language processing (54), emotion perception and regulation (55), social cognition (56), and resilience (57), and functions as a key node in a ventral emotion regulation network (58). Reduced gray matter volume and altered connectivity of the VLPFC have been implicated in suicidality in late-life depression (59). As a target for neuromodulation, it has been suggested the VLPFC may have more direct white matter connections to the SgCC and thus may be a more effective target for modulating SgCC activity (60) compared to the DLPFC (61, 62). Sydnore et al. (63) found that in-scanner rTMS to the VLPFC demonstrated engagement with both the SgCC and the amygdala, with direct white matter connections through the uncinate fasciculus. Likewise, Wu et al. (60) recently reported that positron emission tomography imaging in 19 patients receiving accelerated iTBS to the left DLPFC demonstrated that baseline hypometabolism in the left IFG was associated with clinical improvement, and that more anterolateral targeting results in significant electric field strength in the IFG. As stimulation targets for rTMS have moved more anteriorly and laterally over time, the VLPFC/IFG region may be increasingly exposed to induced electric fields, and may contribute to the increasing efficacy that has been seen with later targets (64).

### 4.4. Importance of electric field modeling in LLD

Our findings highlight the emerging importance of electric field modeling for rTMS for LLD, especially for ensuring adequate dose. The induced electric field from each magnetic pulse engages axon bodies of neurons in gyral crowns and creates lasting physiological effects based on duration, frequency, and field strength (65). If field strength is inadequate, as is seen with rTMS at less than 80% RMT, there may be insufficient neuronal tissue stimulated to create neuroplastic network effects. Likewise, if the strength is excessive, it may lead to adverse effects such as seizure. While a dose–response relationship between electric field magnitude and clinical efficacy has not yet been established, our data suggests that in BA 47, 30 V/m was the threshold below which non-response tended to occur. As only a minority of participants received field intensities at or above this threshold, insufficient dosing may be a further explanation for the lower response/remission rate observed in our study. Placement of the coil closer to this region would increase the dose, as would utilizing electric field modeling to optimize for scalp to cortex distance, coil orientation and local anatomic effects on distribution and strength of *|E|* given specific gyral and sulcal patterns (25, 66, 67). Especially in LLD, where a large proportion of patients may have significant prefrontal atrophy, electric field modeling may enable delivery of prefrontal stimulation at doses of *|E|* that more closely resemble the electric field intensities obtained at motor cortex during threshold determination, and maximize clinical efficacy while maintaining safety limits related to cortical excitability.

Electric field modeling also enables more precise steering of *|E|* to deliver stimulation to the intended target alone. If a coil is not located optimally over the target, the induced field may still “find” the target nearby and achieve the desired clinical effect if greater *|E|* with broader distribution is used. This may explain the benefits seen with deep rTMS for LLD, which is delivered with a H-coil that achieves both deeper and broader stimulation over both hemispheres and may stimulate VLPFC in addition to DLPFC (68). However, increased *|E|* and distribution may have negative implications for focality and anti-therapeutic stimulation of surrounding areas (47). Although generally considered more focal than deep TMS, the spatial distribution of *|E|* with a figure-8 coil over the DLPFC still extends to adjacent cortex regions and can unintentionally stimulate nodes that participate in different networks (67). In our study this tradeoff was observed: while peripheral *|E|* in the neighboring VLPFC was more beneficial for depression outcome, greater field distribution in the posterior DLPFC was less beneficial, confirming what has been posited by connectivity targeting analyzes regarding an anterior–posterior gradient of effect. We believe this argues for use of modeling to ensure *|E|* is directed not only toward the therapeutic target but also away from non-therapeutic or anti-therapeutic regions.

## 5. Limitations

Limitations of this study include its small size, the lack of a sham control group, and a study population skewed toward female Caucasian patients. We did not adjust stimulation intensity for coil to cortex distance in the prefrontal lobe to account for possible frontal lobe atrophy, as all stimulations were intended to be delivered at the maximum allowable safe dose of 120% of RMT. Further work using electric field modeling will likely justify dosing at higher intensities without compromising safety.

## 6. Conclusion

In this open-label, single-arm trial, accelerated fMRI-guided iTBS to the right DLPFC was feasible and effective for treating late-life depression, although not as effective as recent trials of accelerated fMRI-guided TMS to the left DLPFC, possibly due to hemispheric lateralization, age-related effects, treatment resistance, target selection methods, or inadequate dosing. Induced electric field intensity in posterior BA 47 was correlated with antidepressant response, suggesting the importance of generating sufficient electric field strength in anterolateral zones to achieve clinical benefit. Further study of the spatial distribution and magnitude of the induced electric field at the cortical and subcortical level are needed to determine optimal dosing and delivery of rTMS for LLD.

## Data availability statement

The raw data supporting the conclusions of this article will be made available by the authors, without undue reservation.

## Ethics statement

The studies involving human participants were reviewed and approved by University of New Mexico Health Sciences Center Institutional Review Board. The patients/participants provided their written informed consent to participate in this study.

## Author contributions

BG, JY, SH, AP, ED, BH, TO, ML, CA, CO, DB-W, and DQ were responsible for protocol administration and data collection. TJ, JU, and TO were responsible for data analysis and figure design. DQ, DF, JM, CA, BH, JY, and AV were responsible for literature review, study design, data curation, and writing of the manuscript. All authors contributed to the article and approved the submitted version.

## Funding

This study was funded by a UNM Successful Aging Grand Challenge grant and a Mind Research Network Center for Biomedical Research Excellence (CoBRE) Pilot grant (GM122734).

## Conflict of interest

The authors declare that the research was conducted in the absence of any commercial or financial relationships that could be construed as a potential conflict of interest.

Preliminary data from this study was previously presented at the American Association of Geriatric Psychiatry Annual Meeting, Orlando, FL, March 18–21, 2022.

## Publisher’s note

All claims expressed in this article are solely those of the authors and do not necessarily represent those of their affiliated organizations, or those of the publisher, the editors and the reviewers. Any product that may be evaluated in this article, or claim that may be made by its manufacturer, is not guaranteed or endorsed by the publisher.
